# Supplementary material for: Human assembloids recapitulate periportal liver tissue in vitro
Source: Nature. 2025 Dec 17;650(8101):438–49. doi: 10.1038/s41586-025-09884-1 (PMC12893922; doi:10.1038/s41586-025-09884-1)
Supplement: Supplementary file 2 — Reporting Summary [file 41586_2025_9884_MOESM2_ESM.pdf]

nature portfolio

Corresponding author(s): Meritxell Huch

Last updated by author(s): Oct 27, 2025

Reporting Summary

Nature Portfolio wishes to improve the reproducibility of the work that we publish. This form provides structure for consistency and transparency in reporting. For further information on Nature Portfolio policies, see our Editorial Policies and the Editorial Policy Checklist.

Statistics

For all statistical analyses, confirm that the following items are present in the figure legend, table legend, main text, or Methods section.

n/a	Confirmed	
☐	☒	The exact sample size (*n*) for each experimental group/condition, given as a discrete number and unit of measurement
☐	☒	A statement on whether measurements were taken from distinct samples or whether the same sample was measured repeatedly
☐	☒	The statistical test(s) used AND whether they are one- or two-sided<br>*Only common tests should be described solely by name; describe more complex techniques in the Methods section.*
☐	☒	A description of all covariates tested
☐	☒	A description of any assumptions or corrections, such as tests of normality and adjustment for multiple comparisons
☐	☒	A full description of the statistical parameters including central tendency (e.g. means) or other basic estimates (e.g. regression coefficient) AND variation (e.g. standard deviation) or associated estimates of uncertainty (e.g. confidence intervals)
☐	☒	For null hypothesis testing, the test statistic (e.g. *F*, *t*, *r*) with confidence intervals, effect sizes, degrees of freedom and *P* value noted<br>*Give P values as exact values whenever suitable.*
☒	☐	For Bayesian analysis, information on the choice of priors and Markov chain Monte Carlo settings
☒	☐	For hierarchical and complex designs, identification of the appropriate level for tests and full reporting of outcomes
☐	☒	Estimates of effect sizes (e.g. Cohen's *d*, Pearson's *r*), indicating how they were calculated

Our web collection on statistics for biologists contains articles on many of the points above.

Software and code

Policy information about availability of computer code

Data collection

The following software was used for data collection:
Confocal Imaging: Zeiss ZEN blue edition (version 3.6.095.09000)

Brightfield imaging - Leica Application Suite v.4.6.0 (Leica DMIL LED microscope) or v.4.13.0 (Leica M80 stero-microscope) ;

qPCR - QuantStudio™ Design & Analysis 2.7.0 software (ThermoFisher);

Gluconeogenesis, Urea synthesis, Albumin ELISA and bile acid assay - Perkin Elmer Envision 2104 EnVision Manager v.1.13.3009.1401.
Cell Counting- Countess® II FL Automated Cell Counter v.1.0.247
Quantification of xenobiotic metabolism by mass spectrometry - Q Exactive hybrid quadrupole Orbitrap mass spectrometer (ThermoFischerScientific, USA) and TriVersa NanoMate robotic ion source (Advion Interchim Scientific, USA) Chipsoft 8.1.0 software

Data analysis

Confocal imaging data was analysed with FIJI v.2.14.0/1.54f, and segmented using Arivis Vision 4D (Version: 4.1.0. Build: 16702. 20200324) or Motion Tracking v. 8.100.6 (http://motiontracking.mpi-cbg.de). Data analysis of bile canaliculi was performed using a custom script in FIJI, presented in https://github.com/JulienDelpierre/BileCanaliculiSegmentation.
Data presentation and statistical analysis performed with GraphPad Prism v10 (10.3.0) for non-sequencing data.
Source code used for data analysis and visualisation of bulkRNAseq and scRNAseq dataset are available at https://git.mpi-cbg.de/huch_lab/yuan_dawka_kim_liebert_et_al_2025_sequencing and together with processed data on Zenodo (https://doi.org/10.5281/zenodo.17251198).
Spectra were averaged in Xcalubur Qual Browser v.3.0 (ThermoFischerScientific, USA)

For manuscripts utilizing custom algorithms or software that are central to the research but not yet described in published literature, software must be made available to editors and reviewers. We strongly encourage code deposition in a community repository (e.g. GitHub). See the Nature Portfolio guidelines for submitting code & software for further information.

Data

Policy information about availability of data

All manuscripts must include a data availability statement. This statement should provide the following information, where applicable:

- Accession codes, unique identifiers, or web links for publicly available datasets
- A description of any restrictions on data availability
- For clinical datasets or third party data, please ensure that the statement adheres to our policy

The raw single-cell RNA sequencing (dataset id EGAD50000001453) and bulk RNA sequencing (dataset id EGAD50000001454) data (FASTQ files) generated in this study have been deposited in the European Genome-phenome Archive (EGA) under accession number EGAS50000000994. Access to these data is controlled to protect the privacy and identity of study participants. Access requests can be submitted via the EGA website. Requests will be evaluated by the Data Access Committee (DAC) EGAC50000000112 to ensure that proposed data use is consistent with the consent provided by participants. Approved users will be required to sign a Data Access Agreement (DAA) that specifies the permitted uses of the data. A template of the DAA is available at https://edmond.mpg.de/api/access/datafile/250610. Applications are normally reviewed within 10 working days. Count matrices and fully processed data, together with the source code for the sequencing data analysis, are available via Zenodo (https://doi.org/10.5281/zenodo.17251198). Comprehensive lists of differentially expressed genes, gene set enrichment analysis (GSEA) terms, and marker genes are provided in Supplementary Datasets 2–4.

Sources for annotated scRNAseq/snRNAseq count matrices used as reference datasets in the scRNAseq analysis: Data from Andrews et al., 2022, Andrews et al., 2024, and Guiliams et al., 2022 were downloaded from CZ CELLxGENE Discover (census version 2024-07-01)(https://cellxgene.cziscience.com/) using the Python package cellxgene-census (v1.16.2). For these three publications, author annotations of cell types were retrieved by downloading the h5ad files for the respective publications from the CZ CELLxGENE Discover web interface. The data from Brazovskaja et al., 2024 was downloaded from Mendeley Data (https://doi.org/10.17632/yp3txzw64c.1, "allcells_css_annot.rds"). The data from Ramachandran et al., 2019 was downloaded from Edinburgh DataShare (https://datashare.ed.ac.uk/handle/10283/3433, "tissue.rdata"). We provide Jupyter notebooks that perform the data download in our git repository (see code availability statement).

The raw data from mass spectrometry analyses are available at the following repository: https://doi.org/10.17617/3.Z9GMJE.

All other images, qPCR and measurement data are presented within the manuscript.

Research involving human participants, their data, or biological material

Policy information about studies with human participants or human data. See also policy information about sex, gender (identity/presentation), and sexual orientation and race, ethnicity and racism.

Reporting on sex and gender	All patient information regarding sex is provided in Supplementary Table 1 and 2
Reporting on race, ethnicity, or other socially relevant groupings	N/A
Population characteristics	N/A
Recruitment	N/A
Ethics oversight	N/A

Note that full information on the approval of the study protocol must also be provided in the manuscript.

Field-specific reporting

Please select the one below that is the best fit for your research. If you are not sure, read the appropriate sections before making your selection.

☒ Life sciences ☐ Behavioural & social sciences ☐ Ecological, evolutionary & environmental sciences

For a reference copy of the document with all sections, see nature.com/documents/nr-reporting-summary-flat.pdf

Life sciences study design

All studies must disclose on these points even when the disclosure is negative.

Sample size	No statistical methods were used to estimate sample sizes before the study. We based our sample numbers on a combination of previous experience with in vivo and in vitro systems, and standards in the field.
Data exclusions	scRNAseq thresholding involved excluding cells with abnormally low transcripts, and based on high mitochondrial gene content as is standard practice for downstream analysis of this sequencing data. We also excluded doublets to improve stringency, as described in the methods.
Replication	With the exception of scRNAseq due to the nature of the analysis, all experiments were replicated as indicated in the figure legend. For the scRNAseq we did n=4 different donors per condition
Randomization	Experimental procedures always involved processing control and experimental samples in a random order, rather than by condition.
Blinding	Investigators were blinded to the culture conditions for the experiments in Fig1 -3 and associated Extended Data Fig 1-3 and 8-10. For the remainder of the experiments the researchers were not blinded.

Reporting for specific materials, systems and methods

We require information from authors about some types of materials, experimental systems and methods used in many studies. Here, indicate whether each material, system or method listed is relevant to your study. If you are not sure if a list item applies to your research, read the appropriate section before selecting a response.

Materials & experimental systems

n/a	Involved in the study	
☐	☒	Antibodies
☐	☒	Eukaryotic cell lines
☒	☐	Palaeontology and archaeology
☐	☒	Animals and other organisms
☒	☐	Clinical data
☒	☐	Dual use research of concern
☒	☐	Plants

Methods

n/a	Involved in the study	
☒	☐	ChIP-seq
☐	☒	Flow cytometry
☒	☐	MRI-based neuroimaging

Antibodies

Antibodies used	List of antibodies used in this study are provided in Supplementary Dataset 5_reagents_S1.
Validation	All antibodies are commercially available, and were validated for specificity and application by manufacturers listed above. Antibodies were used at concentrations suggested in previous published methodologies or titrated in-house.

Eukaryotic cell lines

Policy information about cell lines and Sex and Gender in Research

Cell line source(s)	The mesenchymal cells and organoid lines are all primary material derived from freshly isolated human livers by investigators in this study. Ethical approval is provided in methods. Five lines (F-PHH1, F-PHH2, F-PHH3, F-PHH4 and F-PHH5) were obtained from Lonza as described in Supplementary Table 2.
Authentication	N/A
Mycoplasma contamination	Mycoplasma contamination was regularly tested on all laboratory lines throughout this study, using MycoAlert® Mycoplasma Detection Kit (Lonza #LT07-118).
Commonly misidentified lines (See ICLAC register)	No ICLAC lines were used in this study.

Animals and other research organisms

Policy information about studies involving animals; ARRIVE guidelines recommended for reporting animal research, and Sex and Gender in Research

Laboratory animals	Male and female Fah-/-/Rag2-/-/Il2rg-/- (FRG) mice were obtained from Jackson Laboratory. Mice were kept under standard husbandry in a pathogen-free environment with a 12 h day/night cycle. Sterile food and water were given ad libitum.
Wild animals	This study did not involve wild animals.
Reporting on sex	Both male and female mice were used in this study.
Field-collected samples	This study did not involve field-collected samples.
Ethics oversight	Mice were maintained in accordance with the Principles of Laboratory Animal Care and the Guide set by the HYU Industry-University Cooperation Foundation.

Note that full information on the approval of the study protocol must also be provided in the manuscript.

Flow Cytometry

Plots

Confirm that:

☒ The axis labels state the marker and fluorochrome used (e.g. CD4-FITC).

☒ The axis scales are clearly visible. Include numbers along axes only for bottom left plot of group (a 'group' is an analysis of identical markers).

☒ All plots are contour plots with outliers or pseudocolor plots.

☐ A numerical value for number of cells or percentage (with statistics) is provided.

Methodology

Sample preparation	Primary human portal fibroblasts culture in DMEM supplemented with 1% HEPES, 1% GlutaMax, 1% Penicillin/Streptomycin, and 20% FBS at 37°C and 5% CO2 were trypsinized using TRYPLE 1x and stained with 1 µg/test Anti-human CD90 (THY1)-APC, 20 µL/test Anti-human CD140a (PDGFRa)-PE, Anti-CD11b/CD31/CD45-PECy7, and EpCAM-Alexa 488 for 30 minutes on ice and washed twice and sorted on a FACS aria, collected in DMEM supplemented with 1% HEPES, 1% GlutaMax, 1% Penicillin/Streptomycin, and 20% FBS and cultured as described in methods at 37°C and 5% CO2.
Instrument	BD FACSAria Fusion
Software	BD-FACS-Diva
Cell population abundance	*Describe the abundance of the relevant cell populations within post-sort fractions, providing details on the purity of the samples and how it was determined.*
Gating strategy	Cells were first gated according to their SSC-A/FSC-A. Then, single cells were identified using FSC-H/FSC-A. The single cell population was then gated according to their EpCAM-FITC+ and CD31,45,11b -PE-Cy7 expression. The negative gate was used to further separate THY1+ vs THY- cells according to the THY-APC positive gating.

☒ Tick this box to confirm that a figure exemplifying the gating strategy is provided in the Supplementary Information.
