# Supplementary material for: Human assembloids recapitulate periportal liver tissue in vitro
Source: Nature. 2025 Dec 17;650(8101):438–49. doi: 10.1038/s41586-025-09884-1 (PMC12893922; doi:10.1038/s41586-025-09884-1)
Supplement: Supplementary file 4 — Comparison with previous research of generation of hepatocytes organoids vs our work. Summary of key parameters in recent studies generating hepatocyte organoids from tissue-derived human or mouse hepatocytes, in comparison to this study. Parameters include cellular origin, donor age, biobank size, culture method, long-term expansion potential, hepatocyte maturity, patient specificity, functional characteristics, and disease modelling capabilities. Only human cell-derived cultures are considered for maturity, precision, functionality, and disease modelling columns. n/a, not applicable. [file 41586_2025_9884_MOESM4_ESM.docx]

Table3 | Comparison with previous research of generation of hepatocytes organoids vs our work

| Refs | Cellular  origin | Age of donor when human hepatocytes | Biobank Patient Counts | Dynamic  culture process | Long-term culture | Maturity* | Precision* (patient specificity) | Modelling liver functional unit* | Functionality*  (tested) | Modelling  Diseases* |
| --- | --- | --- | --- | --- | --- | --- | --- | --- | --- | --- |
| This study | **Fresh patient tissue, adult hepatocytes** | **11-85 yo** | **living biobank 28** | **Expansion and differentiation** | **> 3 months (> 10 passages, the longest culture** **is Passage 28)** | **Similar to freshly isolated hepatocytes** | **Maintain patient-to-patient variation** | **Patient-specific periportal liver assembloids (Modelling part of human liver periportal region, composed of hepatocytes, cholangiocytes and portal mesenchyme)** | **Gluconeogenesis**  **Ureagenesis**  **Individualized drug metabolism** | **Modelling aspects of human biliary fibrosis** |
| Hu et al., 2018^1^ | Mouse adult  and  Human fetal liver cells, | n/a  and  11-20 weeks gestation | n/a | Expansion and differentiation | >3months, mouse  fetal human liver > 16 | Immaturity | n/a | n/a | LDL uptake, | n/a |
| Zhang et al., 2018^2^ | Cryopreserved human hepatocytes | 2 months-56 yo | n/a | 2D expansion and 3D maturation | 2D, > 5 passages | Similar to mature hepatocytes (Incomplete maturity) | n/a | n/a | Ureagenesis, | n/a |
| Peng et al., 2018^3^ | Mouse adult hepatocytes | n/a | n/a | Expansion and differentiation | > 7 months (> 14 passages) | Immaturity | n/a | n/a | n/a | n/a |
| Wesley et al.,  2022^4^ | Human fetal liver cells | 5-17 weeks of gestation | n/a | differentiation | n/a | Fetal | n/a | n/a | n/a | n/a |
| Hendriks et al., 2024^5^ | Human fetal liver cells  Cryopreserved adult human hepatocytes | 10-12 weeks of gestation  0.3-1.7 yo | n/a | Expansion and differentiation | n/a | Immaturity | n/a | n/a | n/a | n/a |
| Dowbaj et al.,  2025^6^ | Mouse adult hepatocytes | n/a | n/a | Expansion and differentiation | > 5 passages | n/a | n/a | Modelling mouse liver periportal region, composed of hepatocytes, cholangiocytes and portal mesenchyme | n/a | Modelling mouse biliary fibrosis |

1 Hu, H. *et al.* Long-Term Expansion of Functional Mouse and Human Hepatocytes as 3D Organoids. *Cell* **175**, 1591-1606 e1519 (2018).

2 Zhang, K. *et al.* In Vitro Expansion of Primary Human Hepatocytes with Efficient Liver Repopulation Capacity. *Cell Stem Cell* **23**, 806-819 e804 (2018).

3 Peng, W. C. *et al.* Inflammatory Cytokine TNFalpha Promotes the Long-Term Expansion of Primary Hepatocytes in 3D Culture. *Cell* **175**, 1607-1619 e1615 (2018).

4 Wesley, B.T., Ross, A.D.B., Muraro, D. et al. Single-cell atlas of human liver development reveals pathways directing hepatic cell fates. Nat Cell Biol 24, 1487–1498 (2022).

5 Hendriks, D. *et al.* Mapping of mitogen and metabolic sensitivity in organoids defines requirements for human hepatocyte growth. *Nat Commun* **15**, 4034 (2024).

6 Dowbaj, A.M., Sljukic, A., Niksic, A. et al. Mouse liver assembloids model periportal architecture and biliary fibrosis. Nature (2025).

Note that this comparison is only with tissue-derived organoids.

n/a, not applicable

*, for this parameters we only consider the cultures derived from human cells
