# Supplementary material for: Human assembloids recapitulate periportal liver tissue in vitro
Source: Nature. 2025 Dec 17;650(8101):438–49. doi: 10.1038/s41586-025-09884-1 (PMC12893922; doi:10.1038/s41586-025-09884-1)
Supplement: Supplementary file 7 — Supplementary Datasets 1-5. [file 41586_2025_9884_MOESM7_ESM.zip › 2024-10-22623C-s7/Supplementary_Dataset-Legends.docx]

**Supplementary Information**

**Human assembloids recapitulate periportal liver tissue *in vitro***

Lei Yuan, Sagarika Dawka, Yohan Kim, Anke Liebert, Fabian Rost, Robert Arnes-Benito, Franziska Baenke, Christina Götz, David Long Hin Tsang, Andrea Schuhmann, Anna Shevchenko, Roberta Rezende de Castro, Seunghee Kim, Aleksandra Sljukic, Anna M. Dowbaj, Andrej Schevchenko, Daniel Seehofer, Dongho Choi, Georg Damm, Daniel E. Stange, Meritxell Huch

**Supplementary Dataset Legends**

**Supplementary Dataset 1: IPA analysis of publicly available datasets.**

This dataset contains the results of Ingenuity Pathway Analysis (IPA) performed on DEGs derived from publicly available transcriptomic and mutational datasets related to hepatocellular carcinoma (HCC). The analysed datasets include comparisons between HCC organoids and liver tissue, post-hepatectomy regenerating hepatocytes, and shared mutations in HCC patient samples, as referenced in the main text and supplementary figures.

- Supplementary Dataset 1-S1. DEG tables provided to IPA analysis. Filtered by |log2Foldchange| >1 and padj < 0.1
- Supplementary Dataset 1-S2. IPA pathway analysis output with -log (adj p-values) of overlap showed. Filtered for terms that adj. p-values < 0.05 in at least 2 comparisons.
- Supplementary Dataset 1-S3. Subset of the Supplementary Dataset 1_S2 used for Extended Data Figure 1d.
- Supplementary Dataset 1-S4. IPA pathway analysis output with activity z-score showed. Filtered for terms that adj. p-values < 0.05 in at least 2 comparisons.
- Supplementary Dataset 1_S5 - Subset of Supplementary Dataset 1_S4 used for Figure 1b.
- Supplementary Dataset 1_S6 - IPA upstream regulator analysis output. Filtered for the molecules belong to the two signalling pathway terms and has an upstream regulator adj-pvalue <0.1 identified as part of the shortlisted pathway.
- Supplementary Dataset 1-S7. Subset of Supplementary Dataset 1_S6 for Extended Data Figure 1e.

**Supplementary Dataset 2: RNAseq analysis.**

This dataset includes bulk RNA-seq analyses across multiple experimental conditions. All pathway analyses include KEGG and Reactome annotations, with p.adjust < 0.05 filtering applied where indicated.

- Supplementary Dataset 2-S1. Meta data for all the samples analysed by bulk RNA seq. Related to Fig 2-4 and Extended Data Fig. 2-4,6.
- Supplementary Dataset 2-S2. Raw counts for all the samples analysed by bulk RNA seq. Related to Fig 2,3 and Extended Data Fig. 2-4,6.
- Supplementary Dataset 2-S3. DEGs between MM+WtnS+TRULI and Primary (fresh isolated PHHs). Related to Extended Data Fig. 2.
- Supplementary Dataset 2-S4. Gene set renrichment analysis (GSEA) MM+WntS+TRULI vs Primary (fresh isolated PHHs). KEGG pathway analysis. Related to Extended Data Fig. 2. Not filtered.
- Supplementary Dataset 2-S5. Gene set renrichment analysis (GSEA) MM+WntS+TRULI vs Primary (fresh isolated PHHs). REACTOME pathway analysis. Related to Extended Data Fig. 2. Not filtered.
- Supplementary Dataset 2-S6. Gene lists from publicly available datasets used for Extended Data Fig. 2,3.
- Supplementary Dataset 2-S7. DEGs between h-HepOrg cultured in DM vs EM. Related to Fig. 2 and Extended Data Fig. 3.
- Supplementary Dataset 2-S8. Gene set renrichment analysis (GSEA) for h-HepOrg cultured in DM vs EM. KEGG pathway analysis. Related to Fig. 2 and Extended Data Fig. 3. Filtered for p.adjust < 0.05.
- Supplementary Dataset 2-S9. Gene set renrichment analysis (GSEA) between h-HepOrg in DM vs EM. REACTOME pathway analysis used for Fig. 2. Filtered for p.adjust < 0.05.
- Supplementary Dataset 2-S10. Gene set renrichment analysis (GSEA) between h-HepOrg in DM vs EM. Go-terms pathway analysis used for Extended Data Fig. 3. Filtered for p.adjust < 0.05.
- Supplementary Dataset 2-S11. Donor specific genes shown in Heatmap in Fig. 3d (ordered like in the heatmap).

**Supplementary Dataset 3: Single-cell RNA sequencing analyses**

This dataset contains scRNA-seq analyses of hepatocytes, cholangiocytes, and mesenchymal cells in liver assembloids, alongside comparative reference data from publicly available human liver tissue. All enrichment results are filtered for adjusted P < 0.05.

- Supplementary Dataset 3-S1. Top1000 Marker gene list of Cholangiocytes in assembloids related to Fig. 4 and Extended Data Fig. 8.
- Supplementary Dataset 3-S2. Top1000 Marker gene list of Hepatocytes in assembloids related to Fig. 4 and Extended Data Fig. 8.
- Supplementary Dataset 3-S3. Top1000 Marker gene list of Portal mesenchyme (PFs) in assembloids related to Fig. 4 and Extended Data Fig. 8.
- Supplementary Dataset 3-S4. Top1000 Marker gene list of Cholangiocytes in human Tissue related to Fig. 4 and Extended Data Fig. 8.
- Supplementary Dataset 3-S5. Top1000 Marker gene list of Hepatocytes in human Tissue related to Fig. 4 and Extended Data Fig. 8.
- Supplementary Dataset 3-S6. Top1000 Marker gene list of Portal mesenchyme (PFs) in human Tissue related to Fig. 4 and Extended Data Fig. 8.
- Supplementary Dataset 3-S7. GSEA for Cholangiocyte cells in assembloids. KEGG and REACTOME pathways. Related to Fig. 4 and Extended Data Fig. 8.
- Supplementary Dataset 3-S8. GSEA for Cholangiocyte cells in human Tissue. KEGG and REACTOME pathways. Related to Fig. 4 and Extended Data Fig. 8.
- Supplementary Dataset 3-S9. GSEA for Hepatocyte cells in assembloids. KEGG and REACTOME pathways. Related to Fig. 4 and Extended Data Fig. 8.
- Supplementary Dataset 3-S10. GSEA for Hepatocyte cells in human Tissue. KEGG and REACTOME pathways. Related to Fig. 4 and Extended Data Fig. 8.
- Supplementary Dataset 3-S11. GSEA for Mesenchymal cells in assembloids. KEGG and REACTOME pathways. Related to Fig. 4 and Extended Data Fig. 8.
- Supplementary Dataset 3-S12. GSEA for Mesenchymal cells in human Tissue. KEGG and REACTOME pathways. Related to Fig. 4 and Extended Data Fig. 8.

**Supplementary Dataset 4: Single-cell RNA sequencing analysis of fibrosis-like assembloids.**

This dataset contains gene set enrichment analyses (GSEA) of cholangiocytes, hepatocytes, and mesenchymal cells from fibrosis-like liver assembloids, compared with cell populations from publicly available fibrotic and healthy human liver tissue. All enrichment results are filtered for adjusted P < 0.05.

- Supplementary Dataset 4-S1. DEGs comparing cholangiocytes between fibrotic-like and homeostatic-like condition. Related to Fig.5 and Extended Data Fig.10.
- Supplementary Dataset 4-S2. DEGs comparing hepatocytes between fibrotic-like and homeostatic-like condition. Related to Fig.5 and Extended Data Fig.10.
- Supplementary Dataset 4-S3. DEGs comparing mesenchyme between fibrotic-like and homeostatic-like condition. Related to Fig.5 and Extended Data Fig.10.
- Supplementary Dataset 4-S4. GSEA enriched in Cholangiocyte cells from fibrotic compared to homeostatic-like conditions. KEGG pathways. Related to Fig.5 and Extended Data Fig.10.
- Supplementary Dataset 4-S5. GSEA enriched in Cholangiocyte cells from fibrotic compared to homeostatic-like conditions. GO-terms. Related to Fig.5 and Extended Data Fig.10.
- Supplementary Dataset 4-S6. GSEA enriched in Cholangiocyte cells from fibrotic compared to homeostatic-like conditions. REACTOME pathways. Related to Fig.5 and Extended Data Fig.10.
- Supplementary Dataset 4-S7. GSEA enriched in Hepatocyte cells from fibrotic compared to homeostatic-like conditions. KEGG pathways. Related to Fig.5 and Extended Data Fig.10.
- Supplementary Dataset 4-S8. GSEA enriched in Hepatocyte cells from fibrotic compared to homeostatic-like conditions. GO-terms. Related to Fig.5 and Extended Data Fig.10.
- Supplementary Dataset 4-S9. GSEA enriched in Hepatocyte cells from fibrotic compared to homeostatic-like conditions. REACTOME pathways. Related to Fig.5 and Extended Data Fig.10.
- Supplementary Dataset 4_S10. GSEA enriched in Mesenchyme cells from fibrotic compared to homeostatic-like conditions. KEGG pathways. Related to Fig.5 and Extended Data Fig.10.
- Supplementary Dataset 4_S11. GSEA enriched in Mesenchyme cells from fibrotic compared to homeostatic-like conditions. GO-terms. Related to Fig.5 and Extended Data Fig.10.
- Supplementary Dataset 4_S12. GSEA enriched in Mesenchyme cells from fibrotic compared to homeostatic-like conditions. REACTOME pathways. Related to Fig.5and Extended Data Fig.10.

**Supplementary Dataset 5: Antibodies, primers, and reagents used in this study.**

Comprehensive list of experimental materials used throughout this study.

- Supplementary Dataset 5-S1. Antibodies with details of target, vendor and catalogue number, working concentration, application, and blocking/permeabilization solution.
- Supplementary Dataset 5-S2. Primers with sequence information.
- Supplementary Dataset 5-S3. Commercial kits with supplier and catalogue number. All reagents were used according to manufacturers’ instructions unless otherwise specified.
